# Tongue Epithelium Cells from shRNA Mediated Transgenic Goat Show High Resistance to Foot and Mouth Disease Virus

**DOI:** 10.1038/srep17897

**Published:** 2015-12-16

**Authors:** Wenting Li, Kejun Wang, Shimeng Kang, Shoulong Deng, Hongbing Han, Ling Lian, Zhengxing Lian

**Affiliations:** 1 Department of Animal Genetics and Breeding, College of Animal Science and Technology, China Agricultural University, Beijing, 100193, China; 2 State Key Laboratory of Stem Cell and Reproductive Biology, Institute of Zoology, Chinese Academy of Sciences, Beijing, 100101, China

## Abstract

Foot and mouth disease induced by foot and mouth disease virus (FMDV) is severe threat to cloven-hoofed domestic animals. The gene 3Dpol in FMDV genome encodes the viral RNA polymerase, a vital element for FMDV replication. In this study, a conserved 3D-7414shRNA targeting FMDV-3Dpol gene was designed and injected into pronuclear embryos to produce the transgenic goats. Sixty-one goats were produced, of which, seven goats positively integrated 3D-7414shRNA. Loss of function assay demonstrated that siRNA effectively knockdown 3Dpol gene in skin epithelium cells of transgenic goats. Subsequently, the tongue epithelium cells from transgenic and non-transgenic goats were infected with FMDV O/YS/CHA/05 strain. A significant decrease of virus titres and virus copy number was observed in cells of transgenic goats compared with that of non-transgenic goats, which indicated that 3D-7414siRNA inhibited FMDV replication by interfering FMDV-3Dpol gene. Furthermore, we found that expression of *TLR7*, *RIG-I* and *TRAF6* was lower in FMDV infected cells from transgenic goats compared to that from non-transgenic goats, which might result from lower virus copy number in transgenic goats’ cells. In conclusion, we successfully produced transgenic goats highly expressing 3D-7414siRNA targeting 3Dpol gene, and the tongue epithelium cells from the transgenic goats showed effective resistance to FMDV.

Goat, as an economic farm animal in the world, can provide ~6.6 million meat product and 1.18 million tonnes fresh furs for human consumption (FAO, 2010). However, goat is susceptible to foot-and-mouth disease (FMD), a highly contagious viral vesicular disease of cloven-hoofed domestic animals, caused by foot-and-mouth disease virus (FMDV)^1^. FMD is notorious for its severely devastating effect on animal health and animal product safety. The global annual cost paid on prevention vaccination and economic loss caused by FMD has been estimated to be ~$5 billion based on FAO statistics. The clinical symptom of goats infected with FMDV is characterized by weight loss, reduced milk yield, lower fertility, and high death rate of infant animals.

FMDV, a single-strand RNA virus, is the typical member of *Aphthovirus* genus of the *Picornaviridae* family. The virus has seven serotypes: O, A, C, SAT1, SAT2, SAT3, and Asia I. Each serotype has multiple subtypes, and up to now, more than 65 subtypes have been found^2^. The genetic variation among subtypes is large and there is nearly no immune protection across the serotypes or subtypes. Currently, the main strategy for preventing FMD is routine vaccination. However, conventional vaccines developed from inactivated FMDV are not effective to all serotypes or subtypes of FMDV and also take the risk of carrying activated virus into healthy animals due to irregular manipulation during vaccines preparation^3^,^4^. Additional vaccines were also exploited, including vaccines developed from live viral vectors^5^, subunit vaccines from FMDV proteins^6^, synthetic peptides from FMDV poly-peptide^6^,^7^, and DNA vaccines from FMDV genome^8^. However, host immune response is not induced until 7 days post-vaccination^9^. Therefore, an efficient, safety and rapid prevention approach for FMDV infection is needed urgently.

Large amount of studies indicate that RNA interference (RNAi) is an effective approach in interfering virus replication^10^. The mechanism of RNAi is to inhibit target gene by expressing endogenous miRNA or homologous small interfering RNA (siRNA)^11^ generated by exogenous short hairpin RNA (shRNA). Currently, RNAi has been widely utilized in battling against FMDV. Virus replication is usually disrupted by transfecting shRNA or shRNA expressing plasmid in RNAi-mediated cell or mouse models^10^,^11^,^12^,^13^. However, groovy RNAi reagents are unstable and also hard to deliver^14^,^15^. Hence, transgenic technology is utilized together with RNAi to edit genome and modify phenotype of animal. RNAi-mediated transgenic models have been established in mouse and a few farm animals. In the transgenic mouse, transfection of shRNA targeting 3D and 2B can effectively inhibit FMDV replication, and the transferred exogenous gene can stably be delivered to offspring^16^,^17^. In transgenic bovine, primary epithelium cells expressing RNAi-LT4 (VP4) or RNAi-LT6 (VP1) shRNA also showed more resistant to FMDV than non-transgenic individuals^18^,^19^.

In this study, we produced transgenic goats with high expression of siRNA targeting 3Dpol gene, and investigated differential disease resistance between transgenic goats (Tg) and non-transgenic goats (NTg) by infecting tongue epithelial cells with FMDV, which provided a model to explore the role of transgenic animals in disease resistance.

## Results

### A highly efficient and conserved shRNA was screened

**Figure 1:**
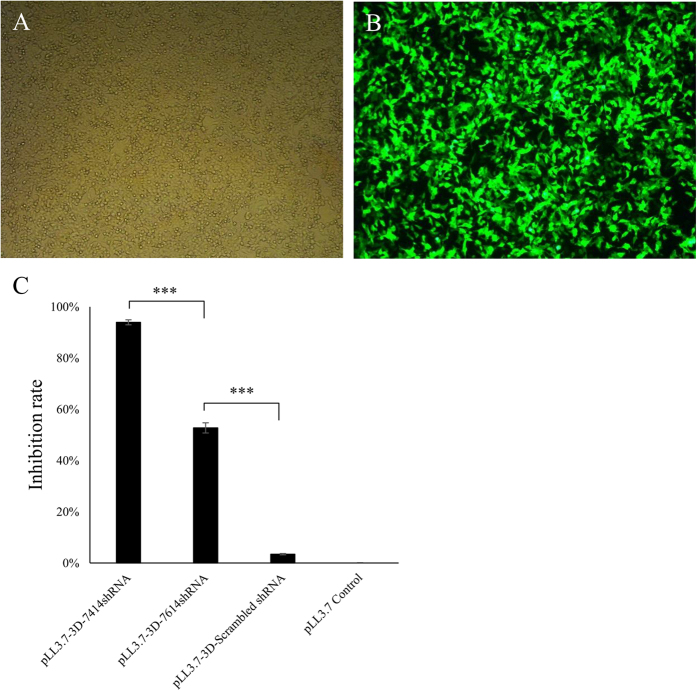
Screening for effective shRNA using Dual-Glo luciferase detection system in 293FT cells. (**A,B**) Bright and dark field of 293FT cells transfected with shRNA expressing vector. (**C**) Inhibition rate of the shRNAs on 3Dpol. The inhibition rate of shRNAs on FMDV 3Dpol was calculated as the formula ([(Renilla/Firefly luciferase activity) _empty group_-(Renilla/Firefly luciferase activity) _shRNA group_]/(Renilla/Firefly luciferase activity) _empty group_.) Data was expressed as means ± SD. ***p < 0.001.

**Figure 2:**
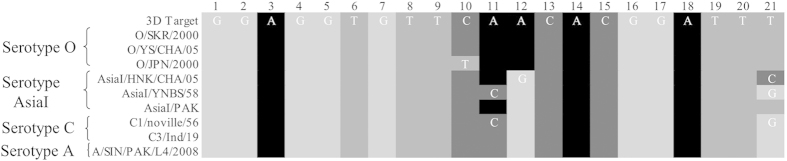
Homology analysis between 3D-7414siRNA and several stereotypes of FMDV 3Dpol sequences.

Two shRNAs, 3D-7414shRNA and 3D-7614shRNA were designed. Dual-Luciferase Reporter Assay System was used to detect the inhibition effects of the two shRNAs on psiCheck2-3Dpol, respectively. The inhibition efficiency of 3D-7414shRNA and 3D-7614shRNA were 93.7% and 52.24%, respectively (Fig. 1). The 3D-7414shRNA was used for the following experiments. The homology between 3D-7414shRNA and nine FMDV subtypes were analysed. The nine FMDV subtypes were derived from four serotypes. Among them, one serotype (Asia I) was originated from Asia and the other three (including O, A and C) were originated from Europe. The results showed that nucleotide sequences of 3D-7414siRNA were highly identical to the target site sequences of nine subtypes. Only four nucleotides, 10th, 11th, 12th, and 21th showed mutations in four serotypes (Fig. 2). The 10th mutation only occurred in O/JPN/2000, and the 12th mutation was different between O serotype and other three serotypes.

### Analysis of 3D-7414shRNA integration and expression in transgenic goats

**Figure 3:**
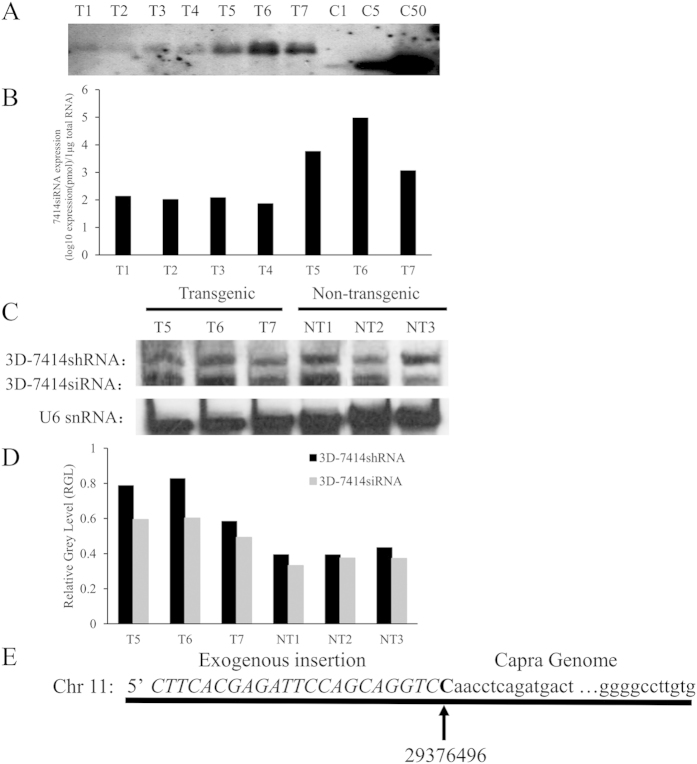
Identification of shRNA insertion and expression analysis. (**A**) Southern blotting for transgenic goat identification. shRNA in transgenic goats was detected. T1, T2, T3, T4, T5, T6 and T7 represented transgenic goats and C1, C5, C50 represented plasmid positive control with one copy, five copies and 50 copies, respectively. (**B**) Absolute quantification of 3D-7414siRNA expression in transgenic goats, T1, T2, T3, T4, T5, T6 and T7 by siRNA-specific qPCR. (**C**) Northern blotting for detecting 3D-7414siRNA transcripts in three transgenic goats T5, T6, T7, and three non-transgenic goats NT1, NT2, and NT3. (**D**) The analysis of grey value of each band in Northern blotting. The grey value was analysed by Bio1D and RGL. The expression of 3D-7414siRNA was calculated relative to U6 snRNA and expressed as ratio. (**E**) Genomic walking analysis for transgenic goat. The arrow points to the position of exogenous fragment inserted.

We microinjected 3D-7414shRNA into pronuclear and produced 61 goats. DNA was isolated from ear of 61 goats, and the exogenous 3D-7414shRNA was successfully detected in seven goats by PCR and southern blotting, in which, three transgenic goats (T5, T6, and T7) showed higher expression of 3D-7414shRNA than the others (T1, T2, T3, and T4) (Fig. 3A). The expression of 3D-7414siRNAs in transgenic goats was quantified by siRNA-specific qPCR. The expression of exogenous 3D-7414siRNA in seven transgenic goat was 1.34 × 10(2), 1.04 × 10(2), 1.18 × 10(2) , 0.72 × 10(2), 5.72 × 10(3), 9.33 × 10(4), and 1.14 × 10(3) pmol/μg (total RNA), respectively (Fig. 3B). Agreed with southern blotting results, T5, T6, and T7 show relative higher expression of exogenous 3D-7414siRNA. The expression of both 3D-7414siRNA and its precursor (3D-7414shRNA) was also analyzed by northern blotting (Fig. 3C). However, we observed similar products in non-transgenic goat (NTg) with the similar length as 3D-7414siRNA and 3D-7414shRNA, respectively, which might be mature endogenous miRNA and its precursor in NTg. The bands of northern blotting were transformed into the relative gray level (normalized to U6 snRNA), which showed that the expression of 3D-7414siRNA and 3D-7414shRNA in transgenic goats was higher than that in non-transgenic goats (Fig. 3D). Furthermore, we detected that the 3D-7414shRNA of T6 was integrated into chromosome 11:29376496 by genomic walking strategy (Fig. 3E).

### The expression analysis of 3D-7414siRNA in epithelium cells by *in situ* hybridization

**Figure 4:**
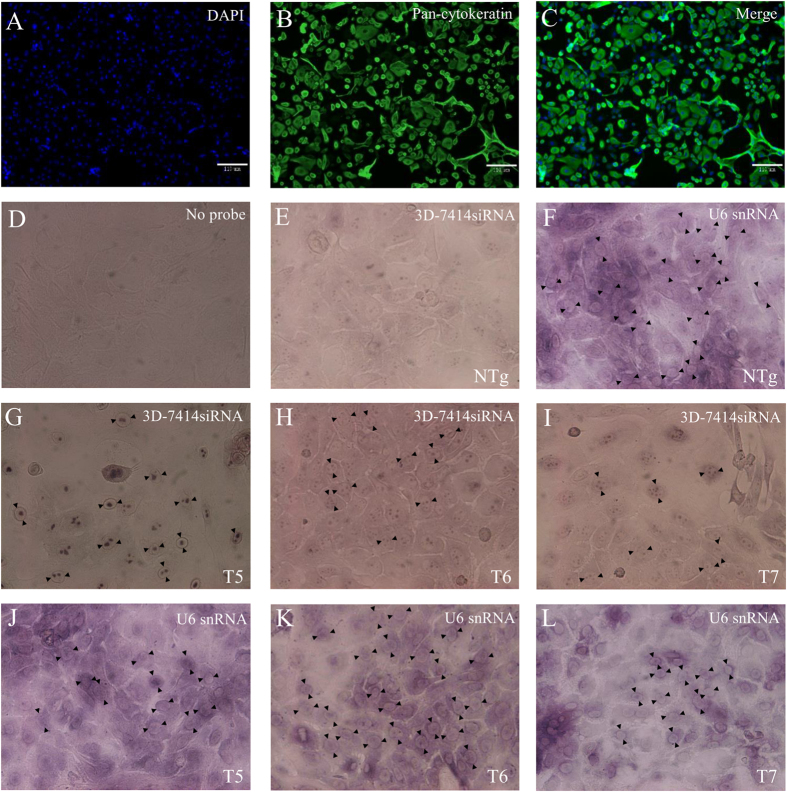
Expression analysis of 3D-7414siRNA in epithelium cells. (**A–C**) Immunofluorescence assay for tongue epithelium cells. The nucleus of epithelium cells were stained with DAPI (**A**). The epithelium cell marker (pan-cytokeratin) flagged with FITC under ImageXpress (**B**). Figure A and B were merged into (**C**). (**D-L**) ISH for identifying 3D-7414siRNA transcripts in epithelium cells. Both 3D-7414siRNA and U6 snRNA transcripts were located around the nucleus. Control group without probe added (**D**). 3D-7414siRNA (**E**) and U6 snRNA (**F**) transcript in NTg were indicated by black arrows. 3D-7414siRNA transcript in T5 (**G**), T6 (**H**) and T7 (**I**) and U6 snRNA transcript in T5 (**J**), T6 (**K**) and T7 (**L**) were pointed out by black arrows.

Primary goat epithelium cells were isolated and identified by immunofluorescence with epithelium cells specific marker (i.e. Pan-cytokeratin). The primary epithelium cells with typical characteristic squamous feature and cytokeratin expression were highlighted by immunofluorescence staining (Fig. 4A–C). The proportion of positive cells estimated by ImageXpress analysis was over 85% in both Tg and NTg. Moreover, *in situ* hybridization (ISH) was conducted to observe the expression of 3D-7414siRNA in epithelium cells of non-transgenic goats and three transgenic goats with U6 snRNA as reference gene. No probe added group acted as control (Fig. 4D). In NTg, no siRNA was detected (Fig. 4E), and only U6 snRNA transcript could be detected (Fig. 4F). Both 3D-7414siRNA transcript and U6 snRNA were observed in T5, T6 and T7, respectively (Fig. 4G–L).

### Loss of function assay demonstrated that siRNA effectively knockdown 3Dpol

**Figure 5:**
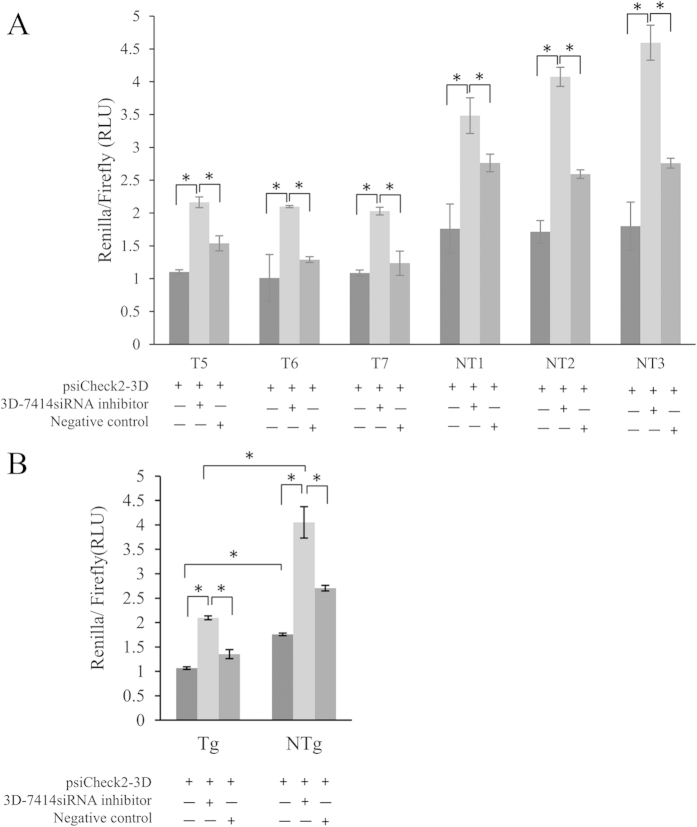
Inhibitory effects of siRNA on 3Dpol gene expression by luciferase reporter assay. (**A**) Inhibitory effects of siRNA on 3Dpol gene expression in epithelium cells from T5, T6, T7, NT1, NT2 and NT3. (**B**) Average inhibitory effects of siRNA on 3Dpol gene in cells from Tg group and NTg group, respectively. Transgenic goats: T5, T6 and T7. Non-transgenic goats: NT1, NT2 and NT3. Data was expressed as mean ± S.E. *p < 0.05.

We transfected the psiCheck2-3Dpol together with 3D-7414siRNA inhibitor or negative control into skin epithelium cells derived from three transgenic goats (T5, T6 and T7) and three non-transgenic goats (NT1, NT2 and NT3). The results for each goat were shown in Fig. 5A. The 3D-7414siRNA inhibitor could significantly rescue 3Dpol gene expression in both three transgenic goats and three non-transgenic goats. Since three transgenic goats sharing a common characteristic of expressing exogenous 3D-7414siRNA, to further analyze the effect of 3D-7414siRNA, we pooled all three transgenic goats together as a group to compare with three non-transgenic goats (Fig. 5B). The average expression of 3Dpol gene measured by relative luminescence ratio was 1.07 ± 0.048 in transgenic goats’ cells transfected with psiCheck2-3D, which was significantly lower than that in NTg group (1.75 ± 0.044). The 3D-7414siRNA inhibitor could strongly rescue the expression of 3Dpol in skin epithelium cells from NTg group, and relative luminescence ratio rose up to 4.05 ± 0.56. In cells from Tg group, the same dose of 3D-7414siRNA-inhibitor also restored the 3Dpol expression, but with relative lower luminescence ratio of 2.09 ± 0.067.

### Tongue epithelium cells of Tg showed lower virus yield than that of NTg after FMDV challenge

**Figure 6:**
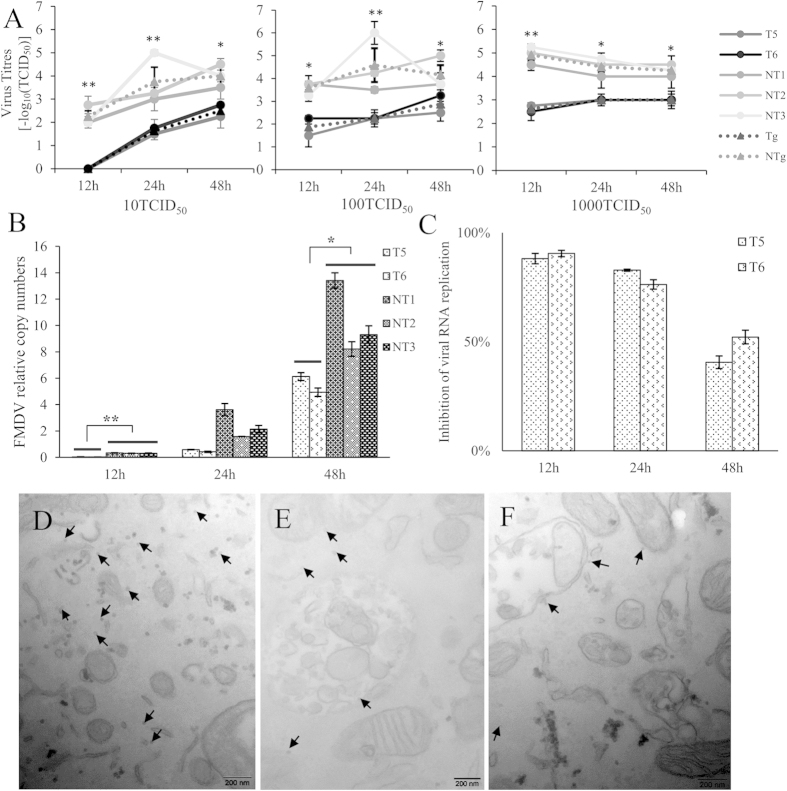
The detection for virus in epithelium cells from Tg and NTg after FMDV infection. (**A**) TCID50 assays for measuring the viral titres in supernatant of T5, T6, NT1, NT2 and NT3 cells. The black and gray dashed lines represented the average viral titres of Tg group (T5 and T6) and NTg group (NT1, NT2 and NT3), respectively. (**B**) The relative viral copy numbers of T5, T6, NT1, NT2 and NT3 cells after 100TCID50 FMDV infection. The goats were classfied into transgenic group, including T5 and T6, and non-transgenic group, including NT1, NT2, and NT3. The viral copy numbers from two groups were compared. (**C**) The inhibition rate in T5 and T6. The rate was calculated as (1-(T5 or T6))/mean of NTg. (**D–F**) Electron microscopy observation of FMDV particles in tongue epithelium cells from NTg (**D**), T5 (**E**), and T6 (**F**). Data was expressed as mean ± S.E. **p < 0.01; *p < 0.05.

Before viral challenge, three Tg (T5, T6 and T7) and three NTg goats (NT1, NT2 and NT3) were diagnosed for complete blood general analysis (Table 1) and blood biochemistry analysis (Table 2). There was no difference between Tg and NTg in those two measurements above. Unfortunately, one of transgenic goats, T7, was dead before viral challenge, therefore only primary tongue epithelium cells from T5, T6, and three NTg were collected and inoculated with 10, 100 and 1000 TCID50 of FMDV O/YS/CHA/05 strain. The virus titres didn’t show any difference between cells from two transgenic goats at 12, 24, and 48 hours post infection (hpi). In order to analyze the difference of virus titres between cells from transgenic and non-transgenic goats, we categorized goats into two groups, with T5 and T6 as transgenic group and NT1, NT2, and NT3 as non-transgenic group. The virus titres were compared between two groups. A significant reduction of virus yield in supernatant was observed in transgenic goats’ cells than that in non-transgenic goats’ cells at all time points and all titres inoculation (Fig. 6A).

To investigate total amount of viral particles in cells, we detected the relative copy number of FMDV in cells inoculated with 100 TCID50 of FMDV by qPCR. The relative viral copy numbers of cells from two transgenic goats (T5 and T6) and three non-transgenic goats (NT1, NT2 and NT3) were individually shown in Fig. 6B. The copy number of virus was increased in both transgenic and non-transgenic goats from 12hpi to 48hpi. There was no difference of viral copy number in cells between T5 and T6 at 12hpi and 24hpi. However, a lower copy number of virus was observed in cells from T6 than that from T5 at 48hpi. We also classified goats into transgenic group, including T5 and T6, and non-transgenic group, including NT1, NT2, and NT3. The average relative copy numbers was 0.034 ± 0.0052 and 0.32 ± 0.024 at 12 hpi; 0.50 ± 0.11 and 2.44 ± 1.6 at 24 hpi, 5.53 ± 0.84 and 10.31 ± 2.9 at 48hpi in tongue epithelium cells from Tg and NTg group, respectively (Fig. 6B).

The copy number of virus in tongue epithelium cells from T5 and T6 was normalized to the mean of viral copy number in NTg to calculate inhibition rate. The inhibition rate in tongue epithelium cells from T5 and T6 was 88.15 ± 2.37%, 90.50 ± 1.48% at 12hpi, 82.85 ± 0.486%, 76.30 ± 2.18% at 24hpi, 40.59 ± 2.91%, 52.15 ± 3.14% at 48hpi, respectively (Fig. 6C). Additionally, electron microscopy was used to clearly observe the virus particles in tongue epithelium cells infected with 100 TCID50 of FMDV. Compared to tongue epithelium cells from NTg (Fig. 6D), a significant reduction of viral particles was detected in T5 (Fig. 6E) and T6 cells (Fig. 6F).

### The expression change of pattern-recognition receptors after FMDV challenge *in vitro*

**Figure 7:**
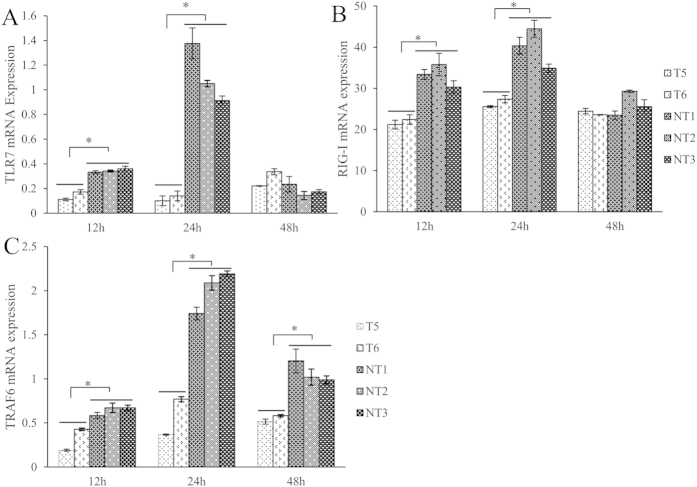
Expression analysis of *TLR7*, *RIG-I*, and *TRAF6* after FMDV challenge by qPCR. (**A**) Differential expression of *TLR7* between tongue epithelium cells from transgenic goats (T5 and T6) and non-transgenic goats (NT1, NT2 and NT3). (**B**) Differential expression of *RIG-I* between tongue epithelium cells from transgenic goats (T5 and T6) and non-transgenic goats (NT1, NT2 and NT3). (**C**) Differential expression of *TRAF6* between tongue epithelium cells from transgenic goats (T5 and T6) and non-transgenic goats (NT1, NT2 and NT3). The differential gene expression from two groups was compared. Data was expressed as mean ± S.E. *p < 0.05.

When epithelium cells were exposed to FMDV, a series of inflammatory responses were initiated to fight against virus replication. In this study, we investigated the expression of two pattern recognition receptors (PRRs), Toll-like receptor 7(*TLR7*) and Retinoic acid-inducible gene-I (*RIG-I*) as well as their downstream gene (Tumour necrosis factor receptor-associated factor 6, *TRAF6*) in epithelium cells between Tg and NTg after treated with 100TCID50 FMDV. The gene expression patterns of the cells from T5, T6, NT1, NT2, and NT3 were presented individually. The comparison of cells from transgenic group (T5 and T6) and that from non-transgenic group (NT1, NT2, NT3) were conducted. Our results showed that expression of TLR7 was lower in epithelium cells from transgenic goats than that from non-transgenic goats at 12 and 24hpi. The average expression in Tg cells was significant lower than the average in NTg cells (Fig. 7A). The expression of another pattern recognition receptor, *RIG-I*, was also lower in Tg cells than that in NTg cells at 12 and 24hpi, not 48hpi (Fig. 7B). *TRAF6* had the same expression pattern with *TLR7* and *RIG-I*, which presented lower expression level in transgenic goats’ cells than that in three non-transgenic cells at 12 and 24. However, the expression of *TRAF6* was also lower in cells of transgenic goats than that of non-transgenic goats at 48hpi (Fig. 7C).

## Discussion

FMDV genome is a single-stranded RNA with the length of 8500nt, containing a unique open reading frame (ORF) coding long poly-peptide, which generates a series of viral structural and functional proteins^2^. Almost all genes in FMDV genome, including VP1, VP2, VP3, VP4, VPg, 2B, 3Dpol, 5′NCR, and 3′NCR, have been ever chosen as RNAi targets^10^,^11^,^13^,^18^,^20^,^21^,^22^,^23^,^24^. Generally, researchers prefer to select highly conserved region as RNAi target, which can interfere more various serotypes. It is reported that identity of FMDV-P3 gene sequence between 11 Asia I subtypes and four O subtypes was over 85%^25^. The 3Dpol as a key part of P3, encodes viral RNA polymerase, and was involved in the synthesis of minus-strand RNA molecule. Besides that, FMDV-3Dpol sequence is highly conserved among several serotypes and subtypes. The homology of nucleotides sequences and amino acids sequences among subtypes is over 90% and 98%, respectively^25^. In this study, the region we chose as siRNA target showed a high homology, ranged from 84% to 100% among nine FMDV subtypes, which presented a high universality across different serotypes.

Recent study discovered that host antiviral RNAi existed in mammals as a self-immune response^26^,^27^. In this study, when we detected the expression of 3D-7414shRNA and 3D-7414siRNA in Tg by northern blotting, we surprisingly found that two products existed in NTg with similar length as shRNA and siRNA (Fig. 3B). However, there was no product but U6 detected in NTg by stem-loop specific primer using siRNA-specific qPCR. The divergence of results from northern blotting and qPCR might be explained by the difference of specificity or sensitivity of two methods. The results indicated that exogenous 3D-7414siRNA was only presented in Tg, but analogous products with similar length and sequences existed in NTg which might be endogenous miRNA. Moreover, in loss of function assay, we also observed that the siRNA inhibitor could rescue the 3Dpol expression in NTg (Fig. 5), which was an additional evidence for existence of endogenous miRNA targeting 3Dpol.

Before infecting tongue epithelium cells with FMDV, goats were diagnosed for complete blood general analysis and blood biochemistry, and no obvious difference was found between Tg and NTg. It indicated that expression of 3D-7414siRNA was innoxious and without negative effect for goat. After epithelium cells inoculated with FMDV in 10, 100 or 1000TCID50, a significant reduction of virus yield in supernatant and viral relative copy number in cells was observed in Tg cell compared to NTg cells. Collectively, a successful transgenic goat model was established which suppressed FMDV replication and spread effectively.

A well-known single strand RNA recognized receptor, *TLR7*, was regarded as a transmitter to activate immune cells when small viral compounds invasion^28^. *RIG-I* was another important PRR which recognizes RNA viruses and triggers the inflammatory response to defence against microorganism invasion. FMDV is a single-stranded RNA virus, but its recognition receptor in goat epithelium cell remained unclear. It was reported that both uncomplexed virus and immune complexed virus stimulated dendritic cell via *TLR7* pathway^29^. Zhou *et al*. (2014) reported that mice immunized with *TLR7* ligands showed a markedly enhanced resistance to FMDV^30^. Kato *et al*. (2006) also reported that *RIG-I* might be a recognition receptor of FMDV and participate in regulating host immunity against FMDV^31^. In this study, we found a relative lower expression of *TLR7* and *RIG-I* mRNA was presented in Tg cells compared with NTg cells. As a downstream gene of both *TLR7* and *RIG-I* pathway, *TRAF6* showed similar expression pattern with *RIG-I* and *TLR7* at 12 and 24hpi. The consistent lower expression of these three genes in Tg cells could be explained by less viral particles load in Tg cells. However, the expression pattern of *TRAF6* was not the same as that of *TLR7* and *RIG-I* at 48hpi. As a downstream gene of inflammation response pathway, *TRAF6* was also affected by several signal pathways, including lipopolysaccharides, interleukin-1, and tumour necrosis factor^32^. Therefore the expression pattern of *TRAF6* was a consequence of multiple factors interaction.

In summary, we successfully produced transgenic goats which highly expressed 3D-7414siRNA targeting 3Dpol gene in FMDV genome. Subsequent experiments proved that tongue epithelium cells from transgenic goats could effectively inhibit virus replication. Our results laid a foundation for investigating disease resistance of transgenic animal model.

## Materials and Methods

### Animals

All the procedures of transgenic goat production and sample collection strictly followed the protocols approved by the Animal Welfare Committee of China Agricultural University (Permit Number: XK662) and this study was carried out in strict accordance with the guidelines and regulations established by this committee.

### Construction of shRNA recombinant vectors

**Figure 8:**
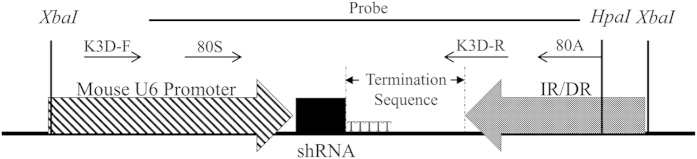
Schematic diagram of insert part of expressing vector. It included mouse U6 promoter, shRNA, and shRNA termination sequences. IR/DR represented a hand of sleeping beauty transposon. PCR primers (K3D-F and K3D-R; 80S and 80A) and southern blotting probe were indicated. *HpaI* and *XbaI* were restriction enzyme cutting sites.

Two candidate shRNAs, 3D-7414shRNA and 7614shRNA, targeting 3Dpol gene (Genbank accession: HQ632768.1) were designed by BLOCK-iT™ RNAi Designer (http://rnaidesigner.lifetechnologies.com/rnaiexpress). The sequences for them were as follows: 3D-7414shRNA (5′-GGAGGTGTTCAACACGGATTTTCA AGAGAAATCCGTGTTGAACACCTCC-3′), 3D-7614shRNA (5′-TGGAGTTGAGCTGGACTC TTACTCAAGAGGTAAGAGTCCAGCTCAACTCC-3′) and a scramble shRNA (5′-AACCTA GCGGAGTTGTACTGCTCAAGAGGCAGTACGGAACTCCGCUAGGTT-3′). The sequences were synthesized by Shanghai Sangon Biological Engineering Technology & Service Co., Ltd. (Sangon, China). The three shRNAs were cloned into pLL3.7 expressing vector (Addgene, USA) to obtain pLL3.7-3D-7414shRNA, pLL3.7-3D-7614shRNA and pLL3.7-scramble shRNA. The expressing vector containing U6 promoter, shRNA, and termination sequences (Fig. 8) was injected into pronuclear embryos.

### Analysis of shRNA inhibition efficiency

FMDV-3Dpol was chemically synthesized based on FMDV sequence (Genbank accession: HQ632768.1) and cloned into psiCheck2 plasmid (Promega, USA) to obtain psiCheck2-3Dpol. Then psiCheck2-3Dpol was co-transfected with pLL3.7-scramble shRNA or pLL3.7-3D-7414shRNA, or pLL3.7-3D-7614shRNA, respectively, into 293FT by Lipofectamine 2000 (Invitrogen, USA). The cells were transfected with pLL3.7 plasmid as empty control. The 293FT cells were grown in Dulbecco’s modified Eagle’s medium (DMEM; Invitrogen, USA) supplemented with 10% fetal bovine serum (FBS; Gibco, USA). All transfection experiments were performed in triplicate. The activities of firefly luciferase and renilla luciferase were detected by Dual-Glo Luciferase Assay System (Promega, USA) at 48h post transfection, and the inhibition rate was calculated by the formula: [(Renilla/Firefly luciferase activity) _empty group_ - (Renilla/Firefly luciferase activity) _shRNA group_]/(Renilla/Firefly luciferase activity) _empty group_. The shRNA with high inhibition efficiency was selected to be injected into pronuclear embryos to produce transgenic goats.

### Identification of shRNA in transgenic goats

Positive transgenic goats was identified by PCR amplification, southern blotting, and *in situ* hybridization (ISH). Briefly, DNA was extracted from the blood and ear tissues using the phenol-chloroform method for PCR amplification and southern blotting, respectively. Two pairs of primers, K3D-F/R and 80S/80A were used for amplification of shRNA, and primer probe-F/R was used to amplify the probe for southern blotting (Table 3). Primary epithelium cells from three transgenic goats (Tg) and three non-transgenic goats (NTg) were cultured in DMEM/F12 (1:1) (Gibco, USA) supplemented with 10% FBS, 0.4 μg/ml Hydrocortisone (Sigma, USA), 100 ng/ml cholera toxin (Sigma, USA), 5 μg/ml Insulin (Sigma, USA), 5 μg/ml transferrin (Sigma, USA) and 10 ng/ml epidermal growth factor (Sigma, USA). The cells were seeded in 12-well plate with 1 × 10(5) cells/well, and were fixed at about 80% confluent by paraformaldehyde for *in situ* hybridization with U6 snRNA as reference and no probe as control. The ISH protocol was followed previous studies^33^,^34^.

### The expression analysis of shRNA in transgenic goats

Northern blotting and siRNA-specific qPCR were performed to quantify the expression of siRNA. For Northern blotting, total RNA was extracted from the skin epithelium cells of three Tg and three NTg by Trizol reagent (Ambion, USA). A total of 15 μg RNA was denatured with 95 °C/10 min and electrophoresis on 15% polyacrylamide gel with 8M urea. 3D-7414siRNA (5′-AAATCCGTGTTGAACACCTCC-3′) and U6 snRNA (5′-CACGAATTTGCGTGTCATCCT- T-3′) locked nucleic acid (LNA) probe labelled with diogoxigenin were purchased from Exiqon (Exiqon, Denmark). The RNA samples were hybridized with the LNA probe as standard protocol^35^ with U6 snRNA as reference. For siRNA-specific qPCR, cDNA was prepared with PrimeScript™ RT reagent Kit (Takara, Japan) using 1 μg total RNA, stem-loop RT primer (500 nM) and ssD1332593108/ssD089261711 primers (RiboBio, China). Genomic walking was finished to detect the insertion site for transgenic goats with multiple nested PCR following standard protocol by Wang *et al*. (2013)^36^.

### Loss-of-function assay in epithelium cell

Epithelium cells (~2 × 10(6)) from three Tg and three NTg were harvested at logarithmic growth phase and transfected with psicheck2-3Dpol. Post-transfected cells were seeded into 96-well-black-wall plate. After 4 h incubation, siRNA inhibitor (Exiqon, Denmark) was transfected into cells with DharmaFECT (Thermo, USA), and firefly and renilla luciferase were detected at 48 h post transfection. All assays were repeated three times with three culture replicates each.

### Analysis of viral titres and gene expression in primary tongue epithelium cells from Tg and NTg after FMDV infection

FMDV challenge experiment was operated in Harbin Veterinary Research Institute with FMDV O/YS/CHA/05 strain. Briefly, primary tongue epithelium cells from two transgenic (T5 and T6) and three non-transgenic goats were cultured in 24-well plates and the following experiments were conducted. The cells were inoculated with different viral doses (0, 10, 100, 1000 TCID50/well). After 1 h of adsorption, the inoculum were removed, and replaced by fresh medium with 2% FBS. The cells were collected for analysis of virus copy number and gene expression at 12, 24, and 48hpi, and the supernatant was collected for evaluating virus titres by using BHK-21 cells. The virus titres in supernatants were expressed as log_10_TCID50/mL calculated by using the Reed-Muench equation. All assays were repeated three times with three culture replicates each. The cells collected at 24hpi were fixed with glutaraldehyde and observed by electron microscopy. Significant differences in the data between groups were tested by one-way ANOVA with a post-hoc test at 5% level.

## Additional Information

**How to cite this article**: Li, W. *et al*. Tongue Epithelium Cells from shRNA Mediated Transgenic Goat Show High Resistance to Foot and Mouth Disease Virus. *Sci. Rep*. **5**, 17897; doi: 10.1038/srep17897 (2015).

## Figures and Tables

**Table 1:** Blood biochemistry parameters of transgenic and non-transgenic goats.

Parameters	Transgenic goats			Non-transgenic goats			P value
	T5	T6	T7	NT1	NT2	NT3	
TP	75.5	87.1	68.7	63.5	70.8	69.309	0.188
AIB	24	24.8	27.3	21.9	26.4	24.1	0.493
Glob	51.5	62.3	41.4	41.6	44.4	45.2	0.262
BUN	6.72	7.5	6.07	10.17	8.37	7.67	0.081
Glu	3.89	3.03	3.92	4.14	4.25	4.07	0.143
K	5.05	5.42	5.08	4.97	4.79	4.74	0.064
Na	148	150	153	148	147	144	0.101
Cl	104	106	108	106	105	104	0.482
CO_2_CP	29.9	22.8	19.2	28.4	26.8	26	0.39
Ca	2.24	2.18	2.53	2.31	2.28	2.2	0.661
Mg	1.1	0.99	1.36	1.14	1.17	1.07	0.847
P	2.8	2.31	2.72	3.18	2.87	2.58	0.311

TP, total protein; AIB, albumin; Glob, globulin; BUN, blood urea nitrogen; Glu, glucose; K, potassium; Na, sodium; Cl, chlorine; CO_2_CP, carbon dioxide combining power; Ca, calcium; Mg, magnesium; P, phosphorus. Statistical analysis completed by student’s t-test with SPSS system. The difference between two groups was analyzed by student’s t-test with SPSS system.

**Table 2:** Complete blood count parameters of transgenic and non-transgenic goats.

Parameters	Transgenic goats			Non-transgenic goats			*P*value
	T5	T6	T7	NT1	NT2	NT3	
WBC	16.71	27.7	20.89	17.49	18.86	26.17	0.836
RBC	4.87	3.71	4.29	4.04	4.12	3.88	0.464
Hb	114	104	117	99	111	108	0.348
HCT	16.5	12.6	14.6	13.7	13.9	13.2	0.446
MCV	33.9	34	34	33.9	33.7	34	0.349
MCH	23.4	28	27.3	24.5	26.9	27.8	0.928
MCHC	691	825	801	723	799	818	0.887
RDW-CV	30.5	29.3	30.4	29.2	29.7	29.4	0.198

WBC, white blood cell count; RBC, red blood cell count; Hb, haemoglobin; HCT, red blood cell specific volume; MCV, erythrocyte mean corpuscular volume; MCH, mean corpuscular haemoglobin; MCHC, mean corpuscular haemoglobin concentration; RDW-CV, red blood cell distribution width- coefficient of variation. The difference between two groups was analyzed by student’s t-test with SPSS system.

**Table 3:** Primers and probe used in this study.

Primer name	Sequence(5′-3′)
80S	AAA CAG CAC AAA AGG AAA CTC ACC C
80A	GAT ACC GTC GAC CTC GAG AAA AAA G
K3D-F	CAT ACA CCT TAG CCA AAT ACA
K3D-R	ACT CGT TTT TCA ACT ACT CCA CA
Probe-F	ATC CGA CGC CGC CAT CTC
Probe-R	ACA ATA GTT TTG GCA AGT CAG TT
LNA probe	AAA UCC GUG UUG AAC ACC UCC
TGF-β1-701F	TGGACATCAACGGGTTCAGT
TGF-β1-967R	GGCAGAAATTGGCGTGGTAG
IL-6 F	GAC ACC ACC CCA AGC AGA CTA
IL-6 R	TGC CAG TGT CTC CTT GCT GTT
IL-10 F	TGC TGG ATG ACT TTA AGG G
IL-10 R	AGG GCA GAA AAC GAT GAC A
TNF-α F	AAC AGG CCT CTG GTT CAG ACA
TNF-α R	CCA TGA GGG CAT TGG CAT AC
TLR7 F	AGCATTTTACTTGTCCCATC
TLR7 R	GCCACTCAAGGACAGAACT
TRAF6-504F	AGAACAGATGCCCAATCACTATGA
TRAF6-728R	GGGACAGAGTCATACGGAGGC
Actin F	AGATGTGGATCAGCAAGCAG
Actin R	CCAATCTCATCTCGTTTTCTG
